# The impact of COVID-19 and associated public health restrictions on trends in police-recorded violence in an English police force area

**DOI:** 10.1186/s12889-023-16366-4

**Published:** 2023-07-28

**Authors:** Carly Lightowlers, Kerri Coomber, Zara Quigg

**Affiliations:** 1 Department of Sociology, Social Policy and Criminology, School of Law and Social Justice, University of Liverpool, Liverpool, UK; 2 Centre for Drug Use, Addictive and Anti-social behaviour Research, School of Psychology, Deakin University, Geelong, Australia; 3 Public Health Institute (PHI), Liverpool John Moores University (LJMU), World Health Organization Collaborating Centre for Violence Prevention, Liverpool, UK

**Keywords:** Violence, Public health, Prevention, Trends, ARIMA modelling, Covid-19 pandemic

## Abstract

**Background:**

The COVID-19 pandemic, and associated public health measures, had a marked impact on a number of health and wellbeing outcomes, including alcohol use and violence. Current literature presents a mixed view of the impact of the pandemic on violence trends. The current study utilises police offence data from a region of northern England to examine the impact of lockdowns, and subsequent relaxation of restrictions, on trends in violent offences.

**Methods:**

Time series analyses using seasonal auto-regressive integrated moving average (SARIMA) modelling was used to investigate the impacts of the COVID-19 public health measures on weekly offence trends from April 1 2018 to March 20 2021. Additionally, pre-pandemic data were used to forecast expected trends had the pandemic not occurred. These expected trends were then compared to actual data to determine if the average levels of violence were outside the forecasted expectations. Overall violence and six subtypes (violence with and without injury, sexual offences including rape, domestic violence, and alcohol-related violent offences) were examined.

**Results:**

Overall, the observed trend in police recorded violent offences demonstrated fluctuating patterns in line with commencement and easing of public health restrictions. That is, offence numbers declined during lockdowns and increased after relaxation of restrictions. However, the majority of observed values fell within the expected range. This broad pattern was also found for subtypes of violent offences.

**Conclusions:**

While violent crime trends demonstrated fluctuations with lockdowns, and subsequent easing of restrictions, these changes were not demonstrably larger than expected trends within this English region, suggesting that a sustained amplification in violence was not observed within this data. However, it is important to acknowledge the high levels of violence reported in this region across the study period, which should be used as a key driver for investing in long-term approaches to violence prevention. Given the extent of unreported violence generally, and that victims/survivors may come into contact with other support services (without reporting to the police), it is vital that policy and practice decisions take a holistic approach, considering a broad range of data sources.

**Supplementary Information:**

The online version contains supplementary material available at 10.1186/s12889-023-16366-4.

## Background

Globally, public health measures in response to COVID-19 (C19) impacted upon a variety of health and wellbeing factors, including alcohol consumption and violence [1–4]. Crime rates, including violence, were also impacted by restrictions on social interaction, access to on-licensed premises and mobility amongst the population [5–8]. Recently, studies have sought to assess short term changes in violence owing to the C19 pandemic [6, 9, 11, 12]. These have mainly focused on whether crime and violence went up or down due to the pandemic and associated public health restrictions. However, findings across these studies, including systematic reviews, have produced mixed results. Internationally, evidence tends to point to a reduction in non-domestic assault due to lockdowns;[7, 13, 14] likely attributable to decreased mobility and offending opportunities. Other literature indicates an increase in domestic assaults after lockdowns were implemented [2, 15]. However, whilst there were increased calls to helplines in a wide range of jurisdictions, evidence on changes in reporting of domestic violence to the police remains yet inconclusive [16, 17]. In the UK, Hohl and Johnson found the “easing of lockdown measures over the summer months had a pronounced impact on domestic abuse coming to police attention”, that is there was a delayed impact “where increased reports and escalating domestic abuse only comes to police attention following the easing of social distancing measures.”[18].

The variation in findings regarding violence trends during C19 is potentially attributable to methodological differences including different data sources, time periods and jurisdictions under study. Several studies have pursued analyses of the pandemic period itself (or a relatively short study period over which changes in violence are being observed), without assessing the changes in violence in the context of broader social trends which predate the pandemic. It is important to take the pre-C19 period into account, as short-term volatility in police recorded violence can be misleading, particularly if more general trends and seasonal fluctuations are not incorporated into modelling.

Police recorded crime data are known to be subject to underreporting and variation owing to changing recording practices and police forces activity over time [18–21]. Moreover, they can suffer from poor quality and incompleteness – not least due to other operational demands on police officers [22]. Nonetheless, police data offer wider offence and population coverage than survey data (e.g., 22 ONS, 2021) [23]. And, whilst operational demands and recording practices necessarily impact upon police data quality, they provide a good indication of (shorter-term) emerging crime trends and the types of cases dealt with by the police [23] and are thought to be a good indicator of demand in respect of night-time economy violence. Moreover, unlike victimisation survey data they are also able to capture more harmful forms of violence such as homicides [20, 24].

Making use of police data from a police force in the North of England, we examined changes in violence – and its subtypes – during the pandemic, taking into account the previous trends over the two years prior to the C19 pandemic. Through analysing police recorded crime data for between 1st April 2018 to 20th March 2021, we aimed to answer the following research questions:

- What impact did C19 restrictions have on violence in a region in England?
- Did C19-related changes in violence vary by type of offence?
- To what extent did C19 impact on the characteristics of violence and when and where they occurred?

The UK had had three national lockdowns on account of the C19 pandemic, the first of which started on the 23rd March 2020. There have also been additional periods in which social interaction and mobility as well as access to hospitality and on-trade alcohol outlets have been curtailed by restrictions aimed at limiting the spread of the virus. Whilst levels and details of restrictions varied considerably over the pandemic period, they can be characterised into seven key phases as outlined by UK Parliament:[25].

- First national lockdown (March to July 2020, P1NL1).
- Minimal lockdown restrictions (July to September 2020, P2MLR).
- Reintroducing restrictions (September to October 2020, P3RR).
- Second national lockdown (November 2020, P4NL2).
- Reintroducing the tier system (December 2020, P5RR).
- Third national lockdown (January to March 2021, P6NL3).
- The steps out of lockdown (March 2021 to present, P7SOLD).

The study region was subjected to all national lockdowns – in which people were ordered to stay at home other than for essential reasons. There were also periods in which tiered regional restrictions were implemented. These saw different local authority areas / regions subject to restrictions based on a tiered system of risk and prevalence /spread of the virus. In each of these periods the study region was subject to assessments of relatively high risk and thus strict restrictions.

The national lockdowns and regional restrictions had marked impact on several behaviours, for example restricting the number of people that could associate together. However, in the context of this study and its focus on violence – and potentially the role of alcohol therein – it is noteworthy that the lockdowns and tiered regional restrictions that comprised the response to the C19 pandemic led to major shifts in alcohol availability from (on-trade) licensed premises (e.g., pubs and restaurants) and night-time economy availability.^1^ Indeed, at times, the hospitality sector was forced to close their doors entirely and limit trading hours on re-opening [26]. Yet, at the same time, alcohol was continuously available from off-trade outlets, including supermarkets, in England and Wales. Indeed “alcohol remained readily available and highly accessible during the initial lockdown period, with [people] making use of online shopping opportunities (including specialist websites) to ensure a well-stocked booze cupboard” [27]. Consequently, during this time the concentration of drinking shifted into the home [28]. It is also the case that during this period nationally alcohol related deaths increased,[29] health inequalities widened[30] and reports of domestic violence rose[11, 30–33]. It is thus important to disaggregate any analysis of violence by subtypes of violence, including those incidents that are domestic in nature and/or that involve alcohol consumption to more accurately assess the impact of the public health restrictions on violence. To the authors’ knowledge, few studies have done so (with only one distinguishing between emergency department visits for violence-related injuries occurring in and outside the home in Cardiff, Wales, early in the pandemic) [34].

## Data and methods

The study deployed the following data, study periods, measures, and analytical approach.

### Data and study periods

Having removed historic crimes (where the lag in reporting was over seven days)^2^, an extract of police recorded crime incident (event) data from a police force (covering a population of ~ 1.4 million) for violent crimes reported between 1st April 2018 to 20th March 2021 (n = 100,135) was subject to analysis to estimate the immediate and delayed impacts of the lockdowns, whilst considering historical trends. Doing so allowed us to distinguish between ‘normal’ fluctuations in violence and changes that could reasonably be attributed to the C19 public health restrictions and so assess the impact of the commencement of the pandemic and associated lockdown measures on trends in violence to be considered in their longer-term context. Given the limited data on the post pandemic period, analysis focused on the changes between the pre-pandemic period and the respective seven stages of lockdown conditions/restrictions over the pandemic as detailed in Brown and Kirk-Wade [25].

### Outcome measures

Weekly aggregate violent crime offences were modelled. In addition, trends in the following violent offence sub-types^3^ were examined:

- Violence With Injury.
- Violence Without Injury.
- Other Sexual Offences.
- Rape.
- Violent incidents flagged as domestic violence.
- Violent incidents flagged as alcohol-related.

### Covariates/controls

Other covariates were selected based on a priori theoretical insights as outlined in the literature review. Temporal changes were key to examining how trends in violence were impacted by the respective lockdown periods and the pre-pandemic periods as outlined by Brown and Kirk-Wade [25]. The time-period in which offences occurred were indicated by an ordinal variable comprising eight categories – the pre-pandemic period and the seven stages of lockdown/restrictions that following during the pandemic period.

### Analytical approach

**Fig. 1:**
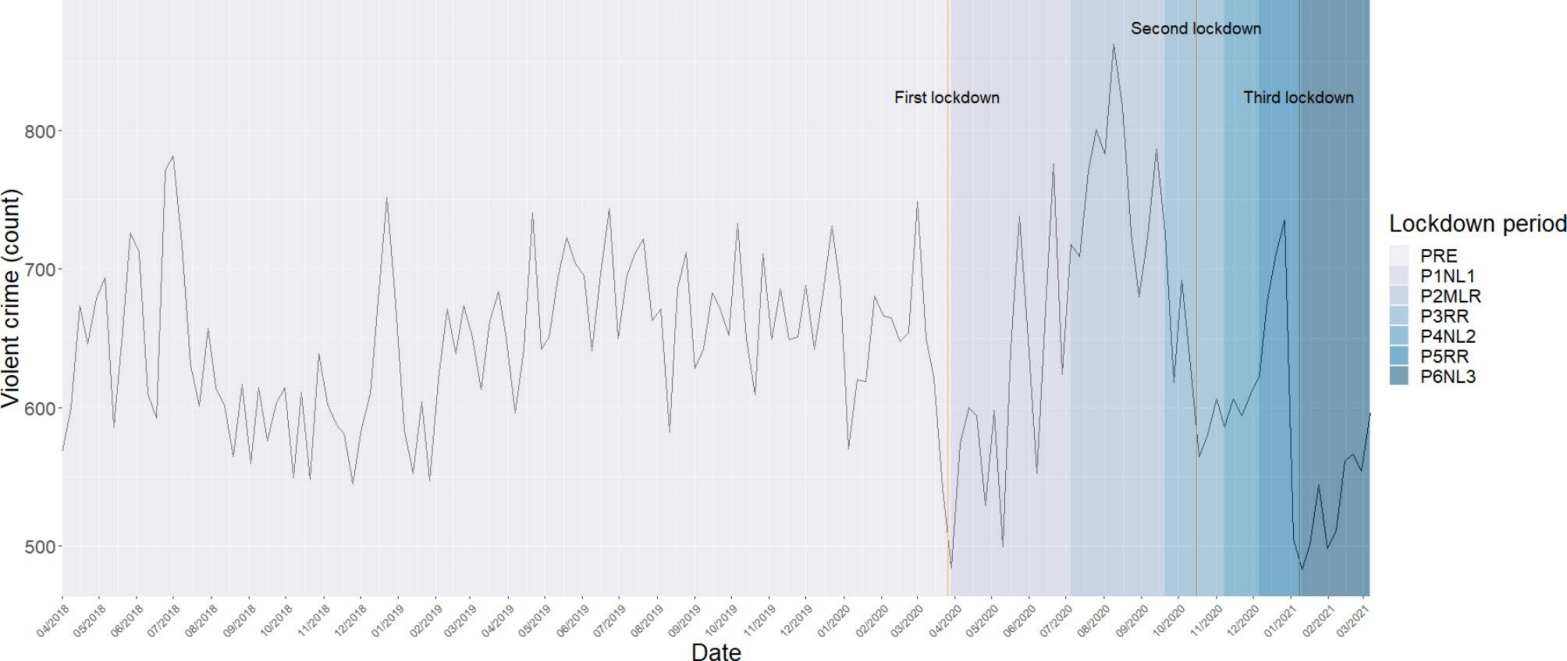
Weekly violent crime counts for police force area, 1st April 2018–20th March 2021. (For ease of visualisation the one observation in the final week in phase P7SOLD has been removed)

Initially descriptive statistics and bivariate tests of significance were run to examine the trends in violence over the C19 pandemic and disaggregating these by key features such as the lockdown period and areas in which they occurred as well as offence types and whether these were domestic in nature or flagged as alcohol-related. The time series was visualised for violence overall (see Fig. 1) as well as its subtypes (See Supplementary Figures I-III).^4^

To build on the exploratory analyses, time series analysis in the form of seasonal auto-regressive integrated moving average (SARIMA) models were run to model the weekly number of violent crimes time series as an aggregate category of crime, but also for six subtypes of violence. Seasonal time series models were used to take historical trends into account and predict what future values might have been in the absence of the pandemic. This longitudinal approach highlights trends that would not be apparent if using a pre/post binary comparison set, as these would not account for ‘normal’ fluctuations in the average levels of violence over time due to changes which are unrelated to the pandemic. This approach thus allows for differentiation between ‘normal’ weekly fluctuations in violence and changes that could reasonably be attributed to the lockdown periods.

SARIMA models remove short term volatility in trends and examine longer term trends by allowing for rolling averages / exponential smoothing. They also allow for seasonal adjustments (i.e., consistent peaks (typically summer and December) or troughs (typically autumn and late winter) in the data series). As “without accounting for these seasonal effects, there is a risk that any observed short-term decline in crime may be overstated or incorrectly attributed as a consequence of COVID-19” [36]. SARIMA models are denoted SARIMA(p,d,q)(P,D,Q,S) where p/P represent the non-seasonal/seasonal autoregressive terms, non-seasonal/seasonal d/D differencing of the data to render it stationary, non-seasonal/ seasonal q/Q the moving average terms and S the time span of a repeating seasonal pattern. Box-Ljung Q tests and Bayesian information criterion (BIC) were used to assess model fit.

These SARIMA models allowed us to forecast likely levels of violent crime in the absence of the pandemic and then compare this to what did happen. That is, by examining whether the actual trend in violence was within the ‘forecasted region’ predicted by the model. The SARIMA models were ran using a subset of training data based on the 104 weekly observations pre-pandemic (1st April 2018 to 20th March 2021) to predict trends in violence in the 51 weeks post pandemic (from March 2020). Therefore, the pre-pandemic data were used to forecast the expected trends had the pandemic not occurred. The actual trend lines were then overlayed to examine the extent to which observed trends over the pandemic (test data) were within the 95% confidence interval for the predicted values. That is, to confirm changes in trends in violence over the course of the pandemic or not. If average levels of violence stayed within the ‘forecasted region’ when the pandemic came into force, this suggests the pandemic did not have an impact on violent crime, whereas if average levels of violence went outside of these forecasts, then the pandemic may have caused changes to trends in violence.

Initially a SARIMA model (base model) was fitted to the violence time series with no covariates. The optimum model settled upon for this time series was a seasonal exponentially smoothed model (also known as an exponentially weighted moving average model or an autoregressive integrated moving average model with no constant term) of the form ARIMA(0,1,1)(0,1,1)[26] errors. The Ljung-Box test suggests no autocorrelation among the residuals (Q* = 11.285, df = 8, p-value = 0.1861), and thus the model was deemed a suitable fit to the time series (BIC = 1454.76).

To test whether the onset of the pandemic had an impact on trends in violent crime beyond the general moving averages (MA) and seasonal adjustments (SMA), the model was rerun to include a binary measure of pandemic onset as a covariate (0 = pre-pandemic, 1 = post-pandemic). This addition did not improve upon the model fit and resulted in an insignificant coefficient suggesting, on aggregate, the trends observed in the pandemic period did not depart significantly from what might have ordinarily been expected/predicted.^5^ The onset of the pandemic was thus not retained as a covariate in further modelling; settling instead on the base model.

Having identified a suitable model specification for the violence time series on aggregate, SARIMA models of the same specification were subsequently run for each type of violence separately; with the (0,1,1)(0,1,1)[26] being a good fit for all series as indicated by the Ljung-Box test of residuals. Models and predictions were also run for violence classified as alcohol-related and domestic in nature.

## Results

Our analyses found that, in the study region, trends in violence fluctuated widely, even before the pandemic (see Fig. 1). This was also the case for each sub-type of violence (See Supplementary Figures I-III).

**Fig. 2:**
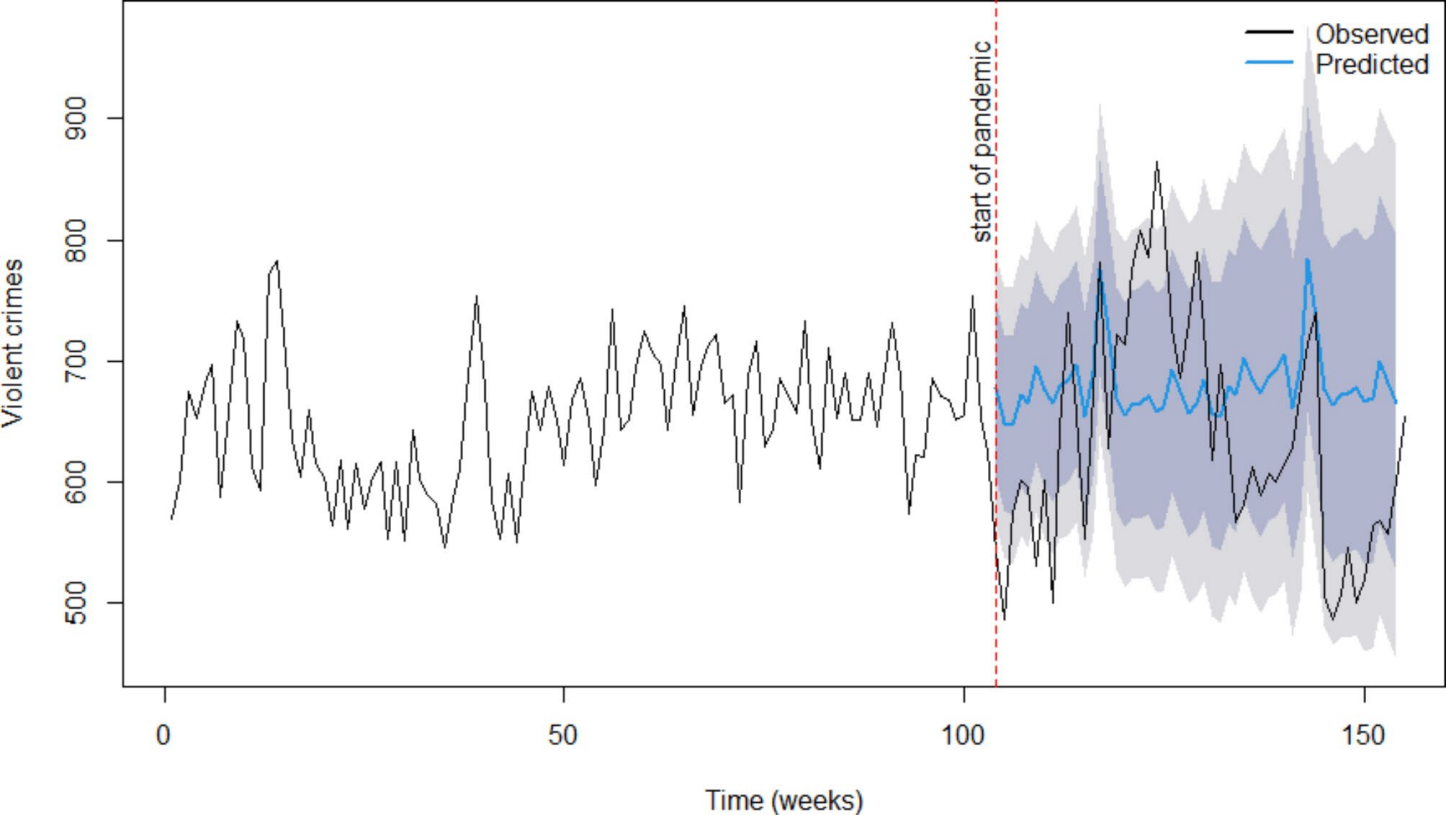
Comparing predictions to observed trends in violence during the pandemic (all violence). The grey shaded area represents the 95% Cis around the forecasted values

SARIMA results presented in Fig. 2 provide the actual compared to forecasted data with 95% confidence intervals (CI). The 95% CI are quite wide; indicative of the fluctuating trends. The observed values during the pandemic seem to be exaggerated values of what was otherwise predicted. However, most observations fell within the predicted range over the course of the pandemic. There were some weeks that exceeded the upper/lower limit of predicted values during the pandemic period. Lower-than-expected values were observed in some weeks early in the pandemic, and some higher-than-expected values observed on releasing restrictions. However, the insignificant effect of the pandemic period when introduced in the model (as well as most values falling within the predicted range) suggests, on aggregate, the trends observed during the pandemic did not depart significantly from what might have ordinarily been expected/predicted.

This broad pattern in overall violent offences was also observed for specific subgroups of violence for the most part (See Supplementary figures IV-IX).

Over the course of the pandemic weekly observations for reports of:

- **violence with injury** were mostly within the predicted range, although when restrictions were lifted, there was one observation that exceeded the upper limit, when restrictions were lifted following the first national lockdown (See Supplementary figure IV).
- **violence without injury** mostly fell within the predicted range, however, early in the pandemic, as well as in subsequent lockdowns, a few observations exceeded the lower confidence limit, indicating temporarily fewer violence without injury incidents early in the pandemic. Although no observations exceeded the upper limit (See Supplementary figure V).
- **rape** mostly fell within the predicted range. However, early in the pandemic, when lockdown measures were introduced, one observation exceeded the lower confidence limit. No observations exceeded the upper limit (See Supplementary figure VI).
- **other sexual offences** mostly fell within the predicted range. However, early in the pandemic, when lockdown measures were introduced, one observation exceeded the lower confidence limit (early in the pandemic, when lockdown measures were initially introduced). Likewise, one observation exceeded the upper confidence limit (on releasing restrictions following the first national lockdown) (See Supplementary figure VII).
- **alcohol-related violence** mostly fell within the predicted range, asides one observation, upon lifting restrictions associated with the first lockdown, which exceeded the upper confidence limit (See Supplementary figure VIII).
- **domestic violence** mostly fell within the predicted range (See Supplementary figure IX). However, early in the pandemic, when lockdown measures were introduced, one observation exceeded the lower confidence limit. Moreover, a seasonal peak exceeded the upper confidence limit in December 2020, just before the third lockdown was introduced.

## Discussion

Consistent with emerging evidence on the impact of lockdown measures on crime,[6, 9–11] our findings show declines in violence in and around the introduction of public health restrictions and rises in violence – specifically sexual and non-fatal violence – as restrictions were eased. Our analysis of the volume of recorded violence revealed that, as in the pre-pandemic period, violence with and without injury made up the bulk of violent offences during the pandemic. Decreases in alcohol-related violence were also observed in and around national lockdowns with increases as restrictions were eased. However, in contrast to other forms, domestic violence increased in and around periods of lockdown / restrictions being introduced (in line with other emerging evidence[2]) and decreased on restrictions being eased.

These findings suggest high levels of violence reported to police in one UK region. Whilst there have been some fluctuations in recorded violence in line with previous studies, when considered within the context of longer term and seasonal trends, on average, levels have remained as expected and the pandemic did not reduce, or increase, violence in this region overall. Whilst this exploratory study does not go as far as to specify and test specific mechanisms, it underscores how sub-setting types of violence can further illuminate specific trends and patterns and so facilitate the testing of further hypotheses relating to specific mechanisms underlying different types of violence (notably sexual, alcohol-related and domestic violence) [37].

It is important to acknowledge that whilst there were peaks and troughs in violence over the pandemic period, with very real implications for victims/survivors and police and multi-agency partner resources and perhaps in part because of responsive / anticipatory police activity, these did not significantly deviate from predicted trends of reported crimes to the police. This evidence suggests that there is volatility in reports of violence in and around specific public health restrictions being introduced, but that longer term trends are likely to remain stable, at least in the first instance. As such our study emphasises the need for long-term data capture and the merit of adopting a longer time series than many studies to date have deployed. Moreover, whilst the observed increases and decreases in violence had implications in the short term (i.e., in terms of the policing response and resource deployment), they should not detract from more strategic longer-term responses to violence in the region or (inter)nationally, including efforts to reduce systemic (gender) inequalities and widespread alcohol availability, the provision of domestic violence services and population level/public health approaches more generally, all of which have been demonstrated as effectively methods for reducing violence [36–40]. Critically, many issues concerning violence in communities pre-date the pandemic; for example, C19 shone a light of a pre-existing domestic abuse crisis (cf., ‘shadow pandemic’) [32].

The data obtained for this study prohibited an examination of homicides (due to low counts) and how changes in violence were distributed across different populations and, of course, may not be representative of other jurisdictions. However, given strong evidence to suggest that violent victimisation, including domestic violence victimisation, is concentrated amongst more impoverished and deprived communities,[37, 40–43] this ought to be a priority area for future data capture and exploration in the region and beyond. Moreover, as our analysis spanned a period of several phases/stages of lockdown in this police force area (and across England more generally) making specific components of lockdowns/public health restrictions and their (lagged) impact on violence difficult to isolate, continued efforts to monitor trends in violence are important. This is vital when considering the complex long-term social, cultural and economic impacts of the pandemic, and how for example, global austerity is likely to increase risks of violence and reduce community assets that protect people from harm [44]. Further, with evidence demonstrating that a large proportion of violence remains hidden, and that during the pandemic both the UK and other countries saw an increase in service utilisation across third sector organisations,[11] such monitoring should include a broad range of data sources to more accurately understand the extent and nature of violence and trends over time. With the emergence of Violence Reduction Units, and subsequent violence surveillance systems in the UK (e.g., https://tiig.ljmu.ac.uk/; Lightowlers et al.[45]) and whole system public health approaches to violence prevention across countries, such systems are emerging and have the potential to make a vital contribution to violence prevention [39, 46.–47].

Of course, police recorded crime data are known to have some key limitations, not least owing to the fact that not all crimes are reported or recorded by the police [48–49] and it can be challenging to identify the precise date on which a crime occurred. In these analyses we removed reports of historical crimes to be able to more confidently comment on trends in crimes occurring during the pandemic and distinct phases thereof (see footnote 2). It is possible this decision will impact upon some forms of violence more so than others – for example, given known lags in the reporting of sexual crimes and potentially where multiple crimes are reported on a single occasion. The latter is also something we are unable to tease out of the data provided, as each entry in the data forms one report of crime, in line with National Crime Reporting Standards [50] which usually prescribes that where there is a sequence of crimes reported in a single incident, the most serious offence is counted. Considering such limitations, police recorded crime data are known to be underestimates of (violent) crime and this should be borne in mind when interpreting our findings.

## Conclusion

While the number of police-recorded violent offences declined with lockdowns, and rose again after restrictions eased, these fluctuations were not outside the expected range had the pandemic not occurred. Thus, within this English region, our study suggests a sustained amplification in violence was not observed within these police data. However, it is important to acknowledge the high levels of violence reported in this region across the study period more generally, which should be used as a key driver for investing in long-term approaches to violence prevention. Given the extent of unreported violence generally, and that victims/survivors may come into contact with other support services (without reporting to the police), it is vital that policy and practice decisions take a holistic approach, considering a broad range of data sources.

## Electronic supplementary material

Below is the link to the electronic supplementary material.

Supplementary Material 1

## Data Availability

The data that support the findings of this study remains under ownership of the local police force and restrictions apply to the availability of these data, which were used under license for the current study, and so are not publicly available. For further queries about these data and conditions of access please contact the corresponding author.

## Footnotes

1 Restrictions on the opening of pubs, bars and hospitality venues and sometimes permitting alcohol only being sold alongside a main meal or unless operating as a takeaway, drive through, and/or delivery service in the latter stages. For example, in phase 3, meetings of more than six people were not permitted and on-trade alcohol premises were required to shut at 10pm. After the third national lockdown (which ran 4th January till 8th March 2021) easing of restrictions started again as part of the ‘roadmap’ out of lockdown. This included the reopening of hospitality venues in a phased manner from 12th April: first allowing service outside and then (from 17th May) indoors. Nightclubs remained closed throughout the pandemic until 19th July 2021 when all restrictions were lifted.

2 There are many complexities to the data collection practices of police data when it comes to crime dates. Our starting point was to ensure we stayed closed to national reporting standards in the (UK Office for National Statistics) published crime figures and use the recording date as in these. That being said, we thought it useful to incorporate some leeway in this regard owing to reporting delays – in this case week allow for a week beyond this date to capture those incidents that occurred in the pandemic phased itself but where people may have needed extra time to report owing to limitations on their liberties. Allowing longer would have made it challenging to discern between crimes having occurred during a particular phase of lockdown (from the 7/8 interrelated stages thereof) and the impact of restrictions being lifted on reporting practices (i.e., people being allowed out and thus able to seek the assistance of the police to report retrospectively). Whilst there is no perfect solution, this approach was though to offer a balance between being able to assume the crimes both occurred and were reported in the pandemic phase of interest, given considerable right skew of lag in reporting with median lag of zero days (Min 0, max 38,004, median 0, mean 189.6) and upon consultation with analysts in the police force itself. Of course, we duly note that extreme values are nevertheless credible values pertaining to the reporting of historic offences (e.g., sexual assaults).

3 Police data contained a binary indication of whether an incident was flagged as domestic in nature and/or alcohol-related.

4 We present quarterly count data in our descriptive statistics and monthly counts in some figures to simplify interpretation and show trends more clearly, but all statistical models were estimated on weekly count data.

5 Further sensitivity analysis was done by testing the effect of this coefficient for all models ran on subtypes of violence. Its introduction in all such models yielded in the same results.
