# Supplementary material for: The impact of COVID-19 and associated public health restrictions on trends in police-recorded violence in an English police force area
Source: BMC Public Health. 2023 Jul 28;23:1448. doi: 10.1186/s12889-023-16366-4 (PMC10375687; doi:10.1186/s12889-023-16366-4)
Supplement: Supplementary file 1 — Supplementary Material 1 [file 12889_2023_16366_MOESM1_ESM.docx]

**Supplementary materials**

**Violence subtypes time series graphs**

Supplementary Figure I: Number of violent crimes recorded by week by type, 1^st^ April 2018 – 20^th^ March 2021^[[1]](#footnote-1)^


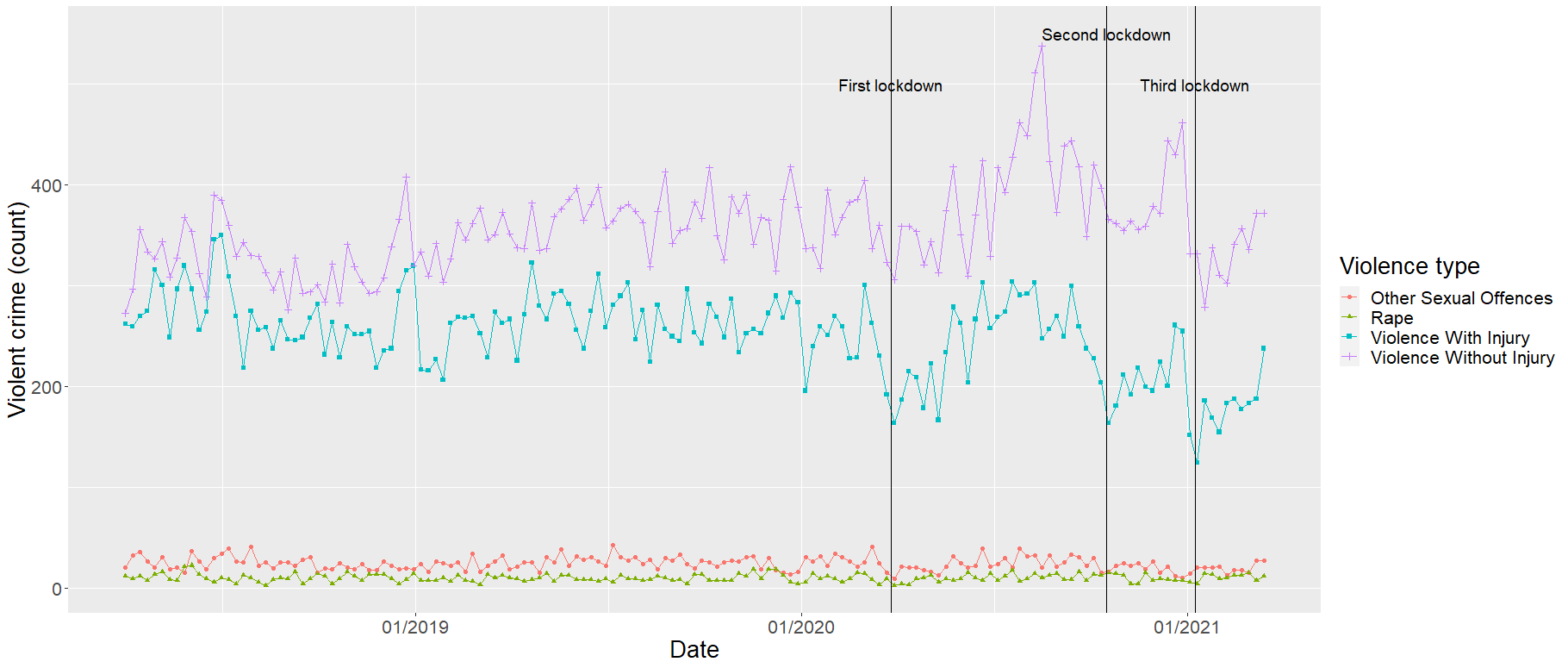


Supplementary Figure II: Number of weekly violent crimes recorded flagged as domestic violence, 1^st^ April 2018 – 20^th^ March 2021^[[2]](#footnote-2)^


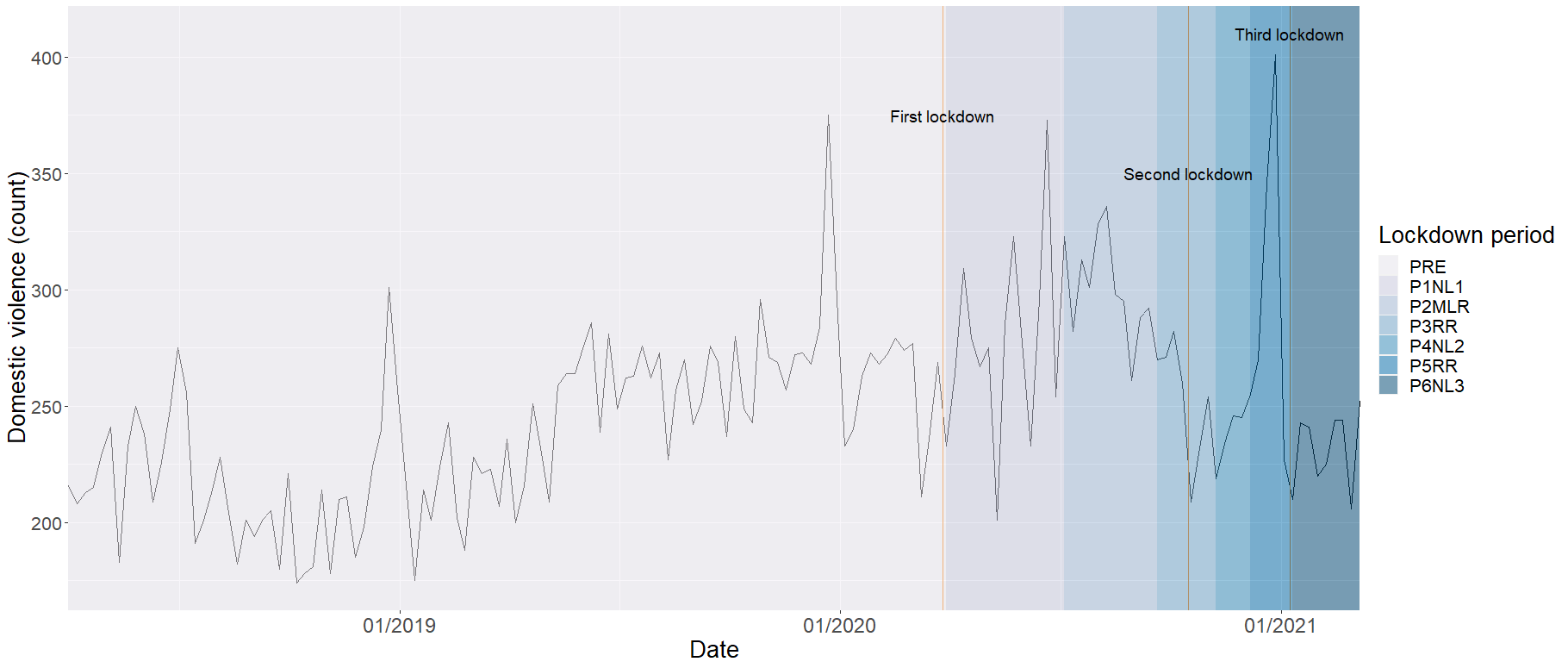


Supplementary Figure III: Number of weekly violent crimes recorded flagged as alcohol related, 1^st^ April 2018 – 20^th^ March 2021^[[3]](#footnote-3)^


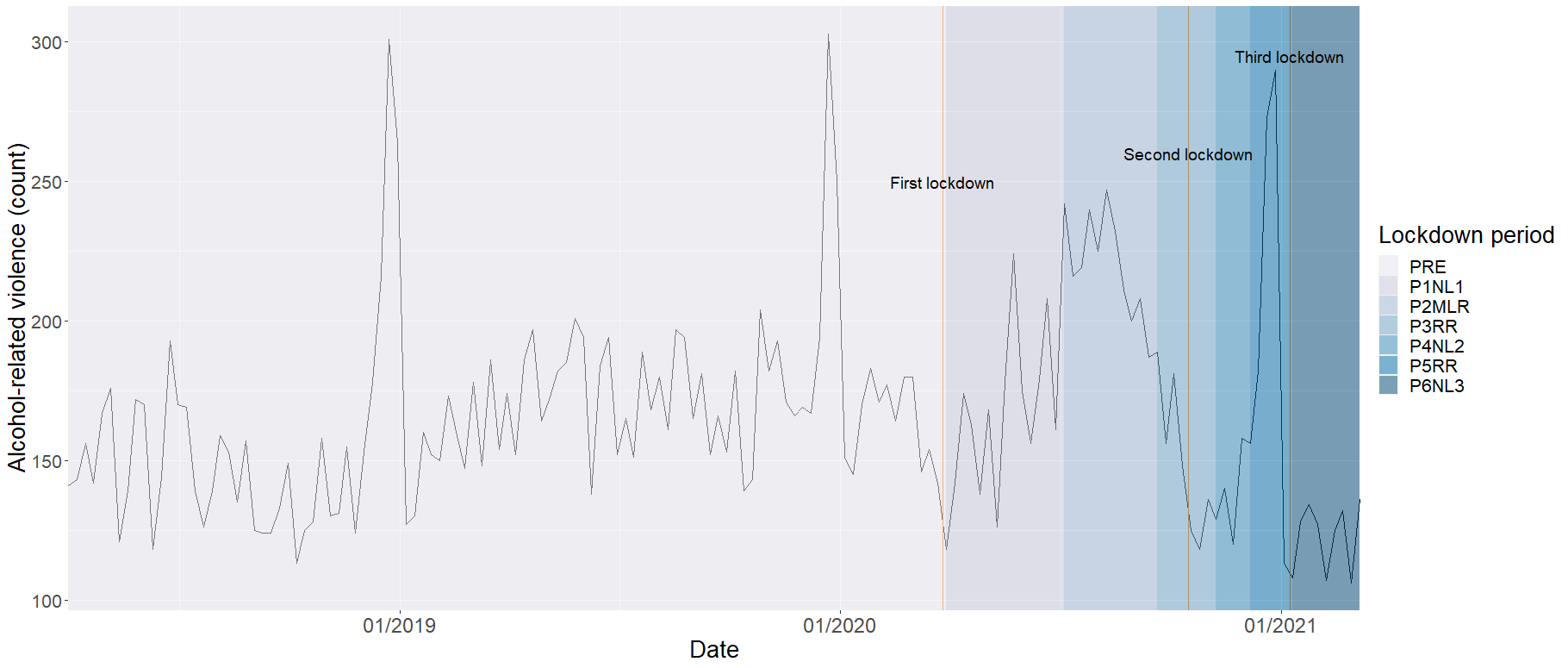


**Violence subtype SARIMA graphs**

Supplementary Figure IV: Comparing predictions to observed trends in violence with injury during the pandemic


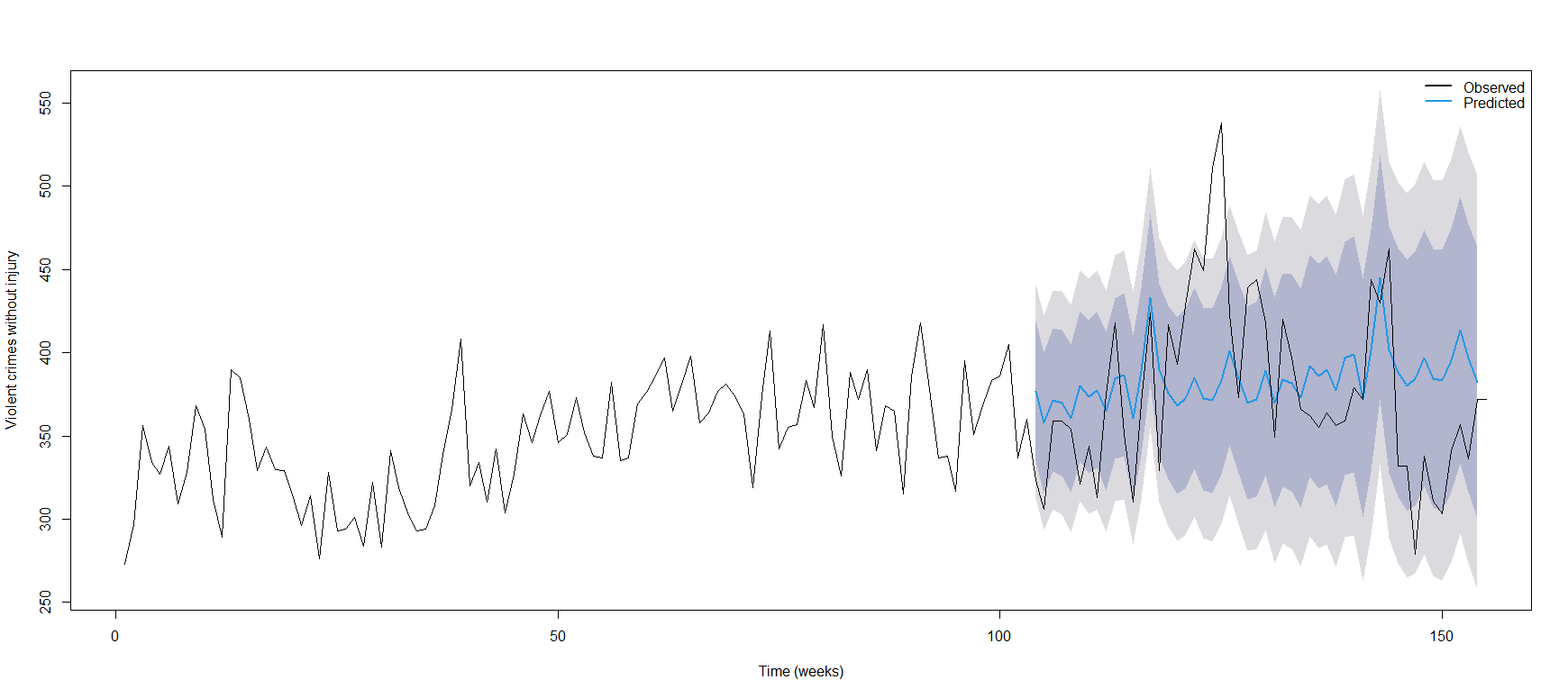


Supplementary Figure V: Comparing predictions to observed trends in violence without injury during the pandemic


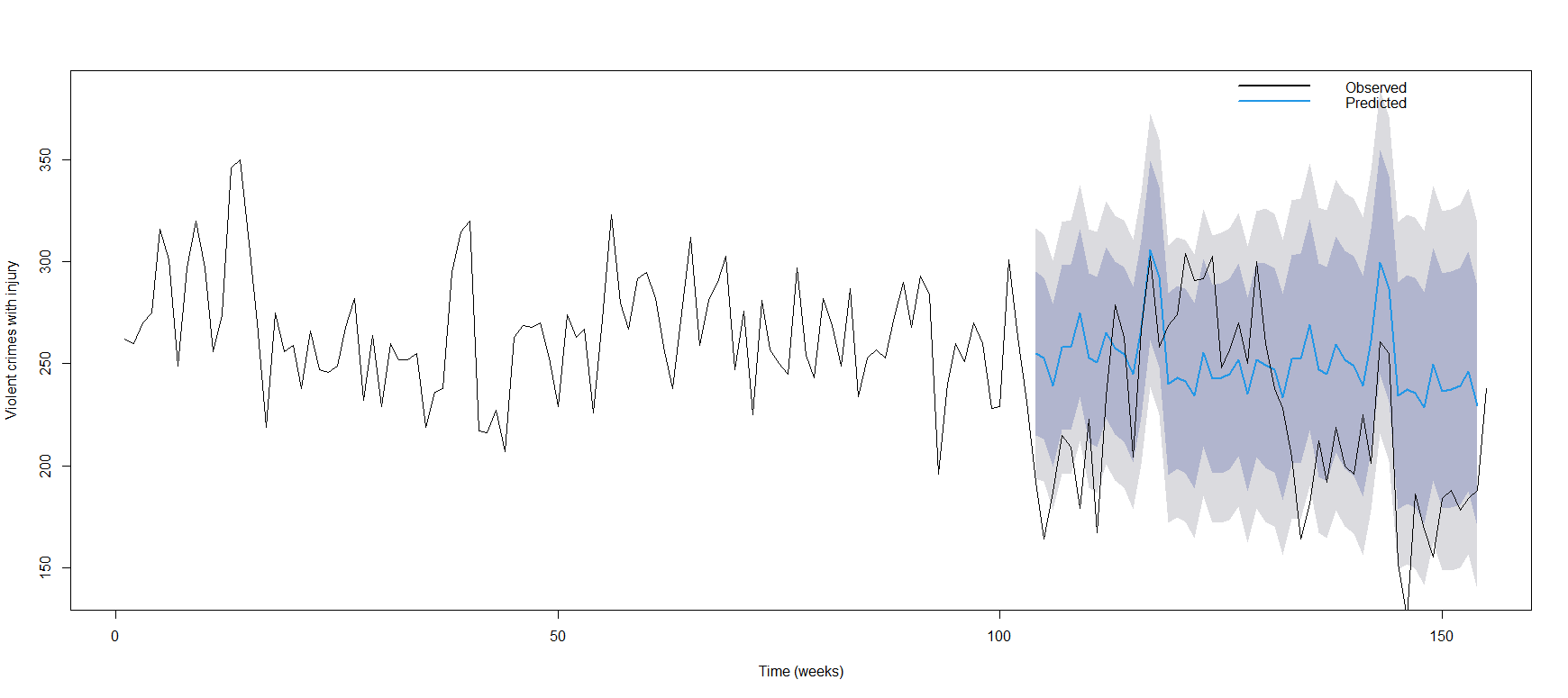


Supplementary Figure VI: Comparing predictions to observed trends in rape during the pandemic


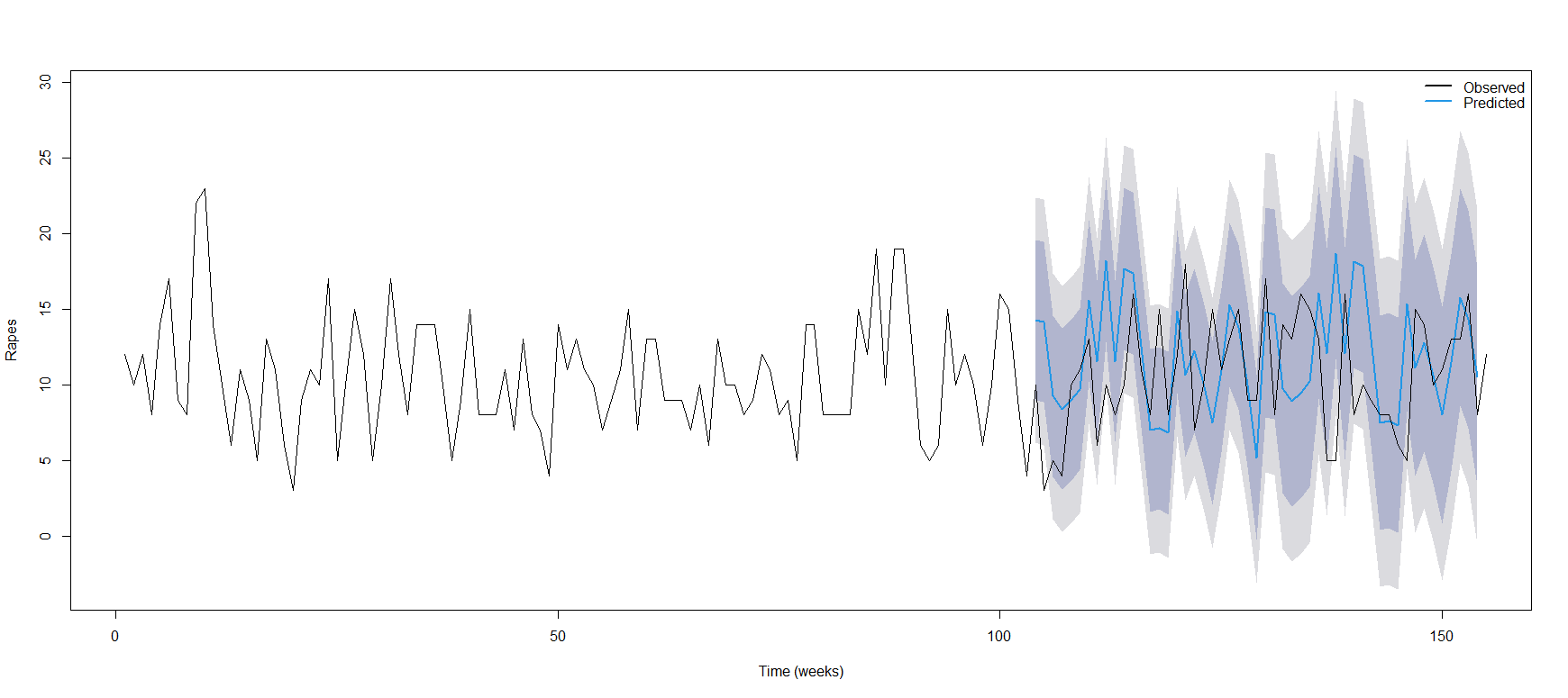


Supplementary Figure VII: Comparing predictions to observed trends in other sexual assault during the pandemic


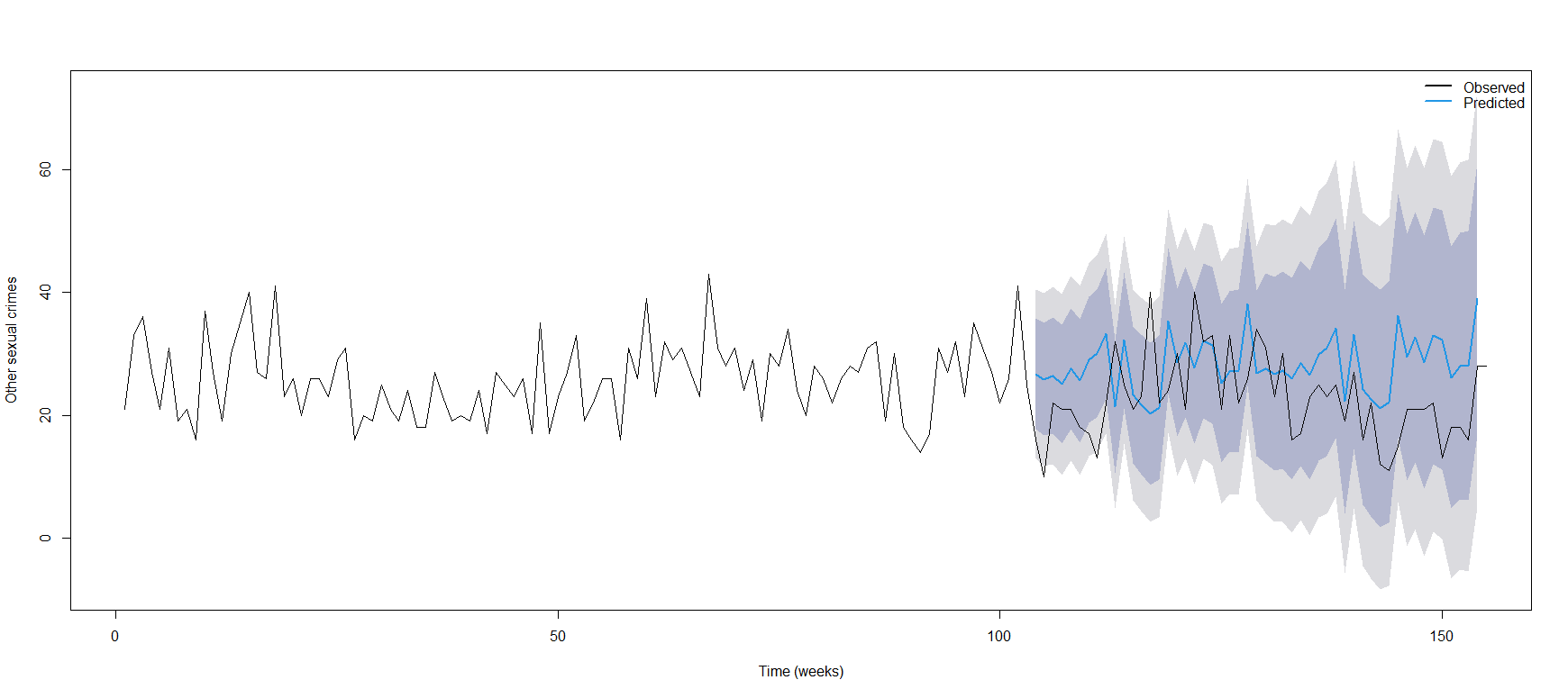


Supplementary Figure VIII: Comparing predictions to observed trends in alcohol-related violence during the pandemic


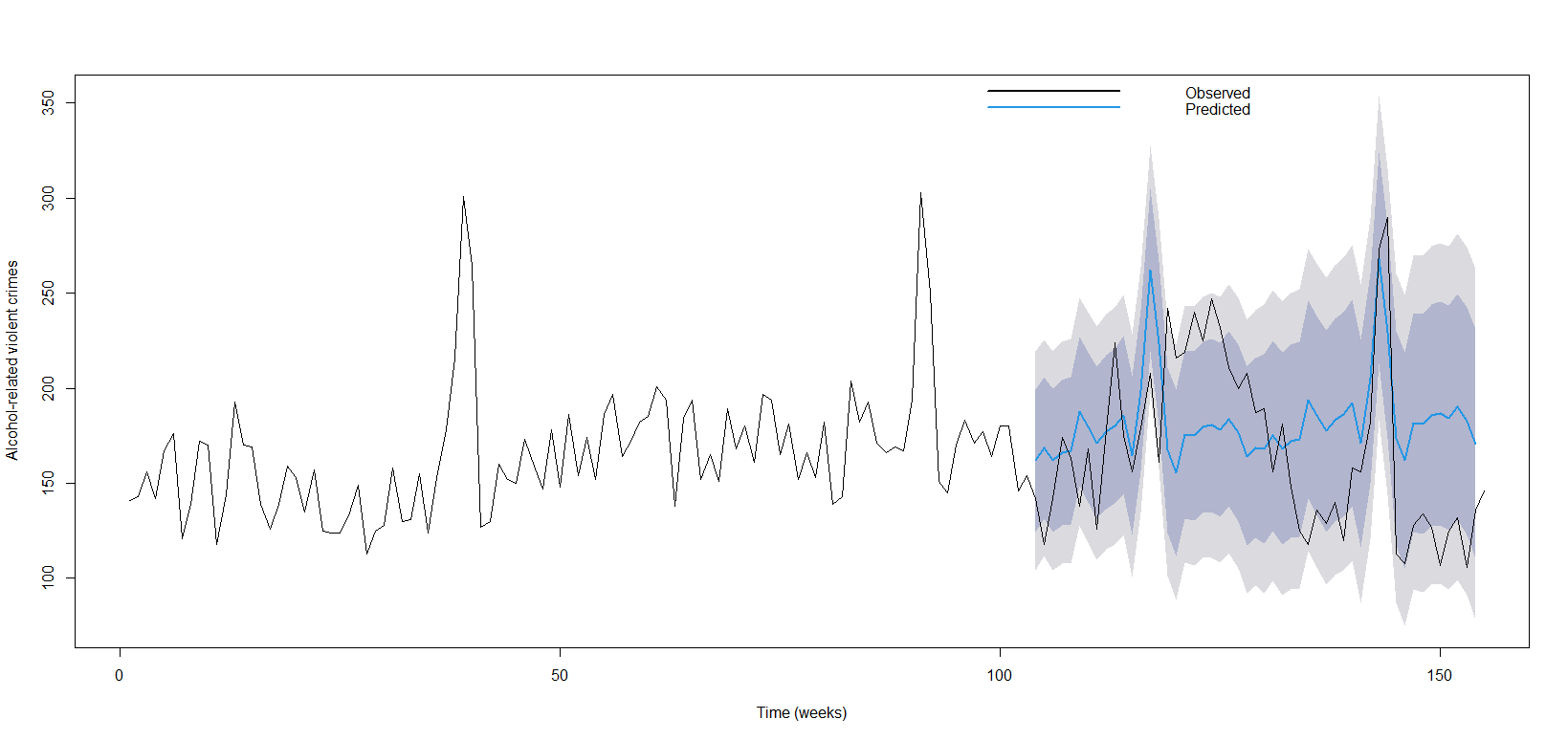


Supplementary Figure IX: Comparing predictions to observed trends in domestic violence during the pandemic

*
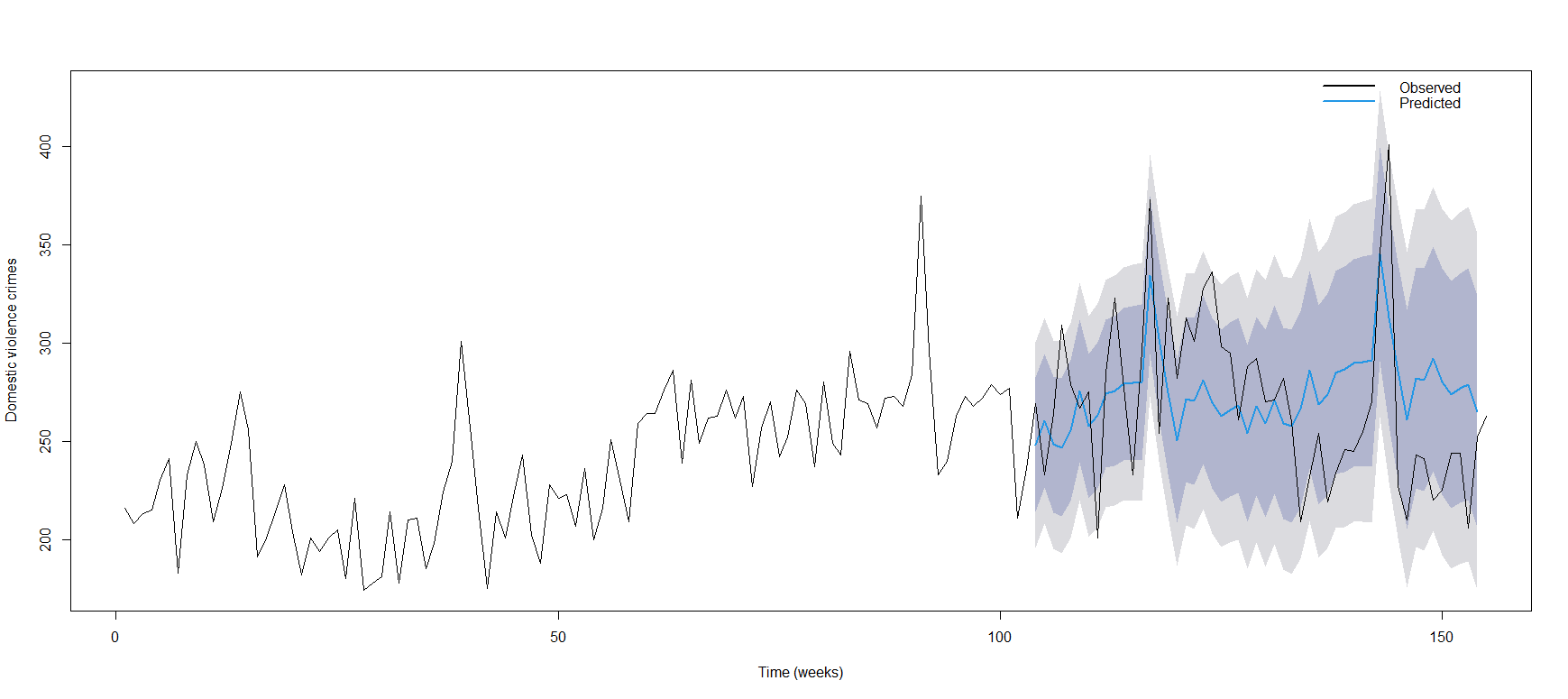
*

1. Given low counts (n<5) of monthly homicides these have not been visualised separately. For ease of visualisation the one observation in the final week in phase P7SOLD has been removed. [↑](#footnote-ref-1)
2. For ease of visualisation the one observation in the final week in phase P7SOLD has been removed. [↑](#footnote-ref-2)
3. For ease of visualisation the one observation in the final week in phase P7SOLD has been removed. [↑](#footnote-ref-3)
